# Directed Evolution of Fungal Laccases

**DOI:** 10.2174/138920211795564322

**Published:** 2011-04

**Authors:** Diana Maté, Eva García-Ruiz, Susana Camarero, Miguel Alcalde

**Affiliations:** 1 Department of Biocatalysis, Institute of Catalysis, CSIC, Cantoblanco, 28049 Madrid, Spain; 2 Centro de Investigaciones Biológicas, CSIC, 28040 Madrid, Spain

**Keywords:** Directed evolution, functional expression, fungal laccases, high-throughput biomolecular screening, *in vivo* DNA recombination, *Saccharomyces cerevisiae*, stability.

## Abstract

Fungal laccases are generalists biocatalysts with potential applications that range from bioremediation to novel green processes. Fuelled by molecular oxygen, these enzymes can act on dozens of molecules of different chemical nature, and with the help of redox mediators, their spectrum of oxidizable substrates is further pushed towards xenobiotic compounds (pesticides, industrial dyes, PAHs), biopolymers (lignin, starch, cellulose) and other complex molecules. In recent years, extraordinary efforts have been made to engineer fungal laccases by directed evolution and semi-rational approaches to improve their functional expression or stability. All these studies have taken advantage of *Saccharomyces cerevisiae* as a heterologous host, not only to secrete the enzyme but also, to emulate the introduction of genetic diversity through *in vivo* DNA recombination. Here, we discuss all these endeavours to convert fungal laccases into valuable biomolecular platforms on which new functions can be tailored by directed evolution.

## 1. LACCASES: GENERAL FEATURES

Laccases (EC 1.10.3.2) are typically extracellular monomeric glycoproteins that belong to the blue multicopper oxidase family (together with ascorbate oxidase, bilirubin oxidase and ceruloplasmin, among others [1-3]). Laccases are considered to be ideal “*green catalysts*” since they are capable of oxidizing a wide variety of compounds in a straightforward manner, using O_2_ from the air and releasing H_2_O as the only by-product [4-6]. These enzymes harbour one paramagnetic T1 copper (producing the beautiful characteristic blue-greenish colour in the oxidized resting state) where the oxidation of the reducing substrate takes place, and a trinuclear T2/T3 copper cluster in which oxygen is reduced to water. The reaction mechanism acts like a battery, storing electrons from the monovalent oxidation of the four reducing substrates required to reduce one molecule of oxygen to two molecules of water [3, 7-9]. Individually, laccases catalyze the oxidation of a wide range of aromatic compounds: ortho and para-diphenols, methoxy-substituted phenols, aromatic amines, benzenothiols, hydroxyindols or syringaldazine. In addition, inorganic/organic metal compounds can also serve as substrates of laccase (e.g. Mn^2+^ can be oxidized to Mn^3+^) [3]. Moreover, the range of reducing substrates can be further expanded to non-phenolic compounds (that are otherwise difficult to oxidize) by including redox mediators from synthetic or natural sources in the so-called laccase-mediator systems. Upon oxidation by laccases, redox mediators oxidize other compounds by non-enzymatic mechanisms, thereby allowing substrates with higher redox potential than laccases to be transformed (*e.g.* non-phenolic lignin dimmers or polycyclic aromatic hydrocarbons, PAHs). Acting as diffusible electron carriers, these mediators can oxidize large polymers such as lignin, cellulose or starch, circumventing the inherent problems of enzyme hindrance normally associated to these substrates [10]. 

Laccases can be found in plants, fungi, bacteria and a few insects. Plant and bacterial laccases belong to the group of low-redox potential laccases (with redox potential at the T1 site [Eº_T1_] around +400 mV), and in particular the latter are of especial interest since they generally display high thermal stability, a quality really appreciated for industrial settings. However, their practical application is still reduced due to the limited range of substrates that they are capable to oxidize. On the other hand, bacterial laccases are useful models for protein engineers, who can take advantage of the ease of manipulation of the bacterial host to carry out rational design or directed evolution. Indeed, recent studies on the directed evolution of laccase CotA from *Bacillus subtilis* has been reported to improve substrate specificity, functional expression and more recently, to use protein surface display to screen mutant libraries [11-13]. 

Fungal laccases have been studied comprehensively because of their strong catalytic capacities and in particular, there have been many studies into directed evolution of fungal laccases in the last few years. This interest initially stemmed from the higher redox potential of the enzyme that permits a broader array of substrates to be oxidised. Indeed, several laccases from white rot fungi (involved in lignin combustion) possess an Eº_T1_ close to +800 mV [2, 5]. Thus, fungal laccases, and especially high-redox potential laccases (HRPLs), may serve: i) to produce 2^nd^ generation biofuels (*i.e.* bioethanol) from lignocellulosic material (*i.e.* laccase and/or laccase-mediator system can oxidize the lignin content of agricultural wastes), or in the manufacture of new products from starch, cellulose and lignin with high added value; ii) in the food industry to process drinks and in bakery products; iii) in the paper industry for pulp-kraft biobleaching, the manufacture of mechanical pulps at low energy cost and to treat effluents; iv) in the textile industry for the remediation of dyes in effluents and textile bleaching (*i.e.* jeans); v) in nanobiotechnology, since laccases belong to the exclusive group of enzymes capable of accepting electrons directly from a cathodic compartment which enables them to be used in the engineering of biosensors (for phenols, oxygen, azides, morphine, codein, catecolamines or flavonoids) with clinical and environmental applications, or in biofuel cells; vi) in the bioremediation through the oxidation of polycyclic aromatic hydrocarbons (PAHs), dioxines, halogenate compounds, phenolic compounds, bencenic derivatives, nitroaromatic compounds and dyes; and vii) in the organic synthesis of complex polymers, drugs, antibiotics and cosmetics [4, 14-17].

There have been many attempts to use rational approaches to engineer fungal laccases over the last couple of decades. In pioneering research carried out by Dr. Xu, several residues in the neighborhood of the catalytic copper ions were subjected to site directed mutagenesis in order to determine what parameters define the catalytic activity and the redox potentials of these enzymes [18-20]. One consequence of these comprehensive structure-function studies was the generation of a collection of mutants with structural perturbations at the T1 copper centre. In addition, directed evolution of fungal laccases (especially HRPLs) has received a recent boost by overcoming the obstacles associated with their functional expression in hosts suitable for *in vitro* evolution experiments [21-24].

## 2. DIRECTED EVOLUTION OF FUNGAL LACCASES

For most of us, directed evolution represents an elegant shortcut to tailor enzymes with improved features. By mimicking the Darwinist algorithm of natural selection through iterative steps of random mutagenesis and/or DNA recombination, the temporal scale of evolution can be collapsed from millions of years into months rather than weeks of bench work [25-27]. In general, it is important to bear in mind three essential aspects when performing laboratory evolution experiments:

- It is necessary to have a reliable and sensitive screening assay to identify the small improvements obtained in each round of evolution, generally 2 to 10-fold improvements per evolutive cycle. In the last years, colorimetric high-throughput screening assays specifically designed for laccase evolution have emerged. All these assays are based on known natural or surrogate substrates of different chemical nature and complexity (from phenols to recalcitrant compounds: ABTS, 2,6 dimethoxy phenol, syringaldazine, iodide, anthracene, or Poly R478 have been used to screen mutant libraries [21, 28-31]). Depending on the approach, the screening assays can be combined in an attempt to enhance several features at once (*e.g.* activity and stability [22] or to avoid the laccase becoming dependent of one specific substrate during evolution [21, 23]).
- Diversity should be generated by random mutagenesis and *in vivo* or *in vitro* DNA recombination protocols [32]. Other approaches such as circular permutation, combinatorial saturation mutagenesis and the combination of rational design with directed evolution are also frequently included in the evolutionary strategy, generally yielding good results [21, 33, 34].
- It must be possible to functionally express the genetic products with the desired traits. Although *Escherichia coli* is the preferred host organism for directed evolution experiments, the broad differences between the eukaryotic expression system of fungal laccases and that of bacteria (codon usage, missing chaperones, post-translational modifications such as glycosylation or the formation of disulphide bridges, and copper uptake) are shortcomings that are not easily overcome. In fact, all attempts to functionally express fungal laccases in bacteria have resulted in misfolding and the formation of inclusion bodies. Alternatively, the secretory machinery of *Saccharomyces cerevisiae *permits post-translational modifications, and it is also an excellent host to carry out laboratory evolution experiments [35, 36].

### 2.1. *Saccharomyces cerevisiae* for Directed Laccase Evolution: A Biomolecular Tool Box for the Generation of Diversity

There are four main reasons to use *S. cerevisiae *for the laboratory evolution of fungal laccases:

- High transformation efficiencies: over 15,000 clones per transformation reaction can be generated.
- Episomal vectors are available that facilitates the recovery of interesting mutants without integration into the genomic DNA as generally happens in yeasts such as *Pichia pastoris*. However, engineering strategies for the evolution of hydroxynitrile lyases in* P. pastoris* have been designed recently, involving the integration of linear expression cassettes to construct and express mutant libraries [37].
- The glycosylation and secretion of laccases avoids the tedious and cumbersome lysis steps required for bacteria. In addition, working with supernatants makes the validation of the screening assay easier since there is much less interference than with complex lysate mixtures.
- And last but not least, a high level of homologous DNA recombination can be achieved, which enables *in vivo* shuffled mutant libraries to be produced or the development of new tools to generate diversity [32, 38, 39].

One of the first directed evolution experiments using *in vivo* DNA shuffling was carried out to engineer a low redox potential peroxidase from *Coprinus cinnereus* with oxidative stability [40]. This pioneering work opened an array of possibilities that led to many research groups (among which we are included) beginning to develop new strategies of DNA recombination [32, 41-43]. Taking advantage of yeast physiology, our group has designed several *in vivo* DNA recombination methods (IVOE, IvAM) with the aim of generating suitable crossover events or varying the mutational bias in the framework of *in vitro* laccase evolution, Fig. (**1**). Sequence splicing by IVOE (*I*n *V*ivo *O*verlap *E*xtension) is a robust and reliable method through which combinatorial saturation mutagenesis, deletion and/or insertion mutagenesis, site-directed recombination or site-directed mutagenesis can be accomplished straightforwardly [32, 39]. The method is based on the engineering of mutagenized primers that contain suitable overhangs, with homologous regions that anneal to each other to generate an autonomously replicating vector containing the mutant gene/s, Fig. (**1A**). This strategy mimics the classical SOE (*S*equence *O*verlap *E*xtension) but it removes several steps, including the *in vitro* cloning. IVOE has been employed to construct mutant libraries for directed evolution of ascomycete and basidiomycete laccases, as well as to carry out semi-rational studies (*i.e.* combinatorial saturation mutagenesis coupled to high-throughput screenings [21, 33]). IvAM (*I*n *v*ivo *A*ssembly of *M*utant libraries) is another approach that has been successfully used to engineer fungal laccases for improved organic co-solvent tolerance, Fig. (**1B**) [38, 44]. Generally, error-prone PCR methods are unsatisfactory because they are associated with a limited and predicted mutational spectrum derived from the intrinsic bias of each DNA-polymerase. To overcome this problem, the mutation biases of different polymerases can be combined by alternating between them in successive rounds of evolution. IvAM has allowed us to explore the laccase sequence space through the *in vivo* DNA shuffling of several mutant libraries with different mutational spectra in a single round of evolution. It is also possible to bring together strategies for *in vitro* and* in vivo* DNA recombination to evolve enzymes in the laboratory. For example, CLERY (*Combinatorial Libraries Enhanced by Recombination in Yeast*) combines *in vitro* and *in vivo* DNA shuffling [45]. In a similar approach, we have combined mutagenic StEP (*S*taggered *E*xtension *P*rocess) with *in vivo* DNA shuffling to evolve ligninolytic peroxidases. There is also an interesting report on how to engineer chimeric fungal laccases from *Trametes *C30 by *in vivo* DNA shuffling [43], where a low redox potential laccase gene was used in all the chimeric libraries to guarantee functional expression. This example constitutes a valuable point of departure for the potential application of the *S. cerevisiae* machinery for laccase chimeragenesis.

### 2.2. Directed Evolution of Ascomycete Laccases

**Fig. (2):**
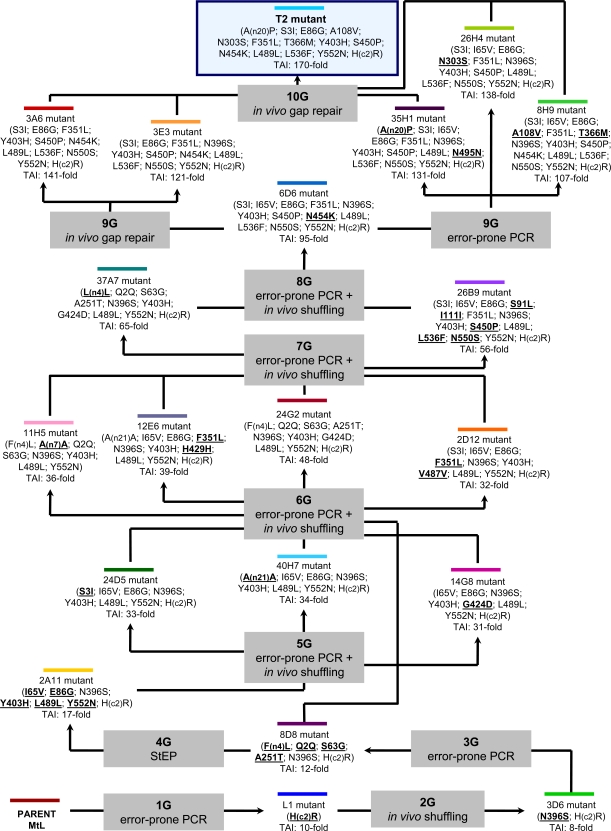
Artificial evolution pathway for MtL in yeast. TAI: total activity improvement over MtL parent type.

The first laccase gene subjected to directed evolution was the *Myceliophthora thermophila* laccase (MtL), a low-medium redox potential ascomycete laccase that is very thermostable (with T_50_ values ~75.6ºC, [41]). In this work, 10 rounds of laboratory evolution were carried out to achieve the strongest functional expression of a laccase in *S. cerevisiae *yet reported (up to 18 mg/L, Fig. **2**). The basic tools for the generation of diversity included error prone PCR, StEP and *in vivo* DNA shuffling. The latter was modified in such a manner that error-prone PCR products were recombined *in vivo* to introduce new mutations in conjunction with recombination, Fig. (**1C**). Furthermore, backcrossing recombination was employed to eliminate neutral mutations. In the final rounds of evolution, PCR and *in vivo* gap repair were used to recombine neighbouring mutations in a site-directed fashion, once again taking advantage of the eukaryotic apparatus (referred to as *in vivo* assembly recombination) which proved extremely useful to eliminate some deleterious mutations. The sequence targeted for directed evolution included the native pre-proleader, as well as the C-terminal tail of the gene that encode for parts of the protein that are cleaved during maturation. The ultimate evolutionary product obtained after screening over 20,000 clones, the T2 mutant, harboured 14 mutations, Fig. (**3A**). The single most beneficial mutation was found in the C-terminal tail, introducing a cleavage site for the *Kex2* protease of the Golgi compartment of *S. cerevisiae*. This mutation probably adjusted the sequence to the different protease specificity of the heterologous host. In a later study, we employed the T2 evolved mutant to study the role of the C-terminal plug of ascomycete laccases. Using combinatorial saturation mutagenesis through IVOE, a direct relationship between the C-terminal plug and a conserved tripeptide in the vicinity of the reducing substrate binding site was determined [33].

Many applications of fungal laccases (bioremediation, lignocellulose processing, organic synthesis, etc.) require high concentrations of organic co-solvents in which laccases may unfold and lose their activity [16]. In another recent study, we used the evolved MtL (T2 mutant) as our point of departure to confer organic co-solvent tolerance [44]. In this work, five rounds of laboratory evolution were carried out to explore 13,000 clones, using IVOE, IvAM and error-prone PCR, in combination with *in vivo* DNA shuffling for library creation. The laboratory evolution strategy was carefully planned according to the following rules:

- Screening was performed (based on the oxidation of ABTS) in the presence of two co-solvents of different chemical nature and polarity in order to identify variants active in both co-solvents. The ultimate goal was to provide promiscuity in other water-solvent mixtures.
- To improve the activity and stability in organic co-solvents, only those mutant *hits* that retained their stability and that were more active were considered as candidates for further cycles of evolution.
- The extreme selective pressure, increasing the concentrations of organic co-solvents from 20 % in the first cycles up to 60 % in the last round, further drove the evolution.
- The spectro-electrochemistry of the variants was studied in depth to assess the influence of the evolution process on the transit of electrons through the laccase structure.

The final mutant (R2 variant) was fairly active and stable at concentrations as high as 50 % of organic co-solvents, (retaining between 20-30 % of its activity in aqueous solution). Moreover, this variant showed promiscuity for different organic co-solvents (DMSO, DMF, DMA, acetonitrile, acetone, ethanol, methanol). Significantly, the spectro-electrochemical features of the enzyme were slightly modified (*i.e.* Eº_T1_ and copper geometries), although these changes did not exert any important differences in either its kinetics or stability. R2 harboured four mutations in the mature protein (E182K, S280N, L429N and N552H), as well as two mutations at the C-terminal tail (G8D, E9K). The mutations at the C-terminal tail seemed to affect protein folding, whereas some mutations at the surface of the mature laccase established new interactions, either through salt bridges or hydrogen bonds. These novel interactions were reflected in the structural reinforcement of the regions amenable to denaturation under harsh conditions.

### 2.3. Directed Evolution of HRPLs

The past successes with MtL evolution cannot easily be translated to their HRPL counterparts, in part because MtL is an ascomycete laccase that facilitates its functional expression in *S. cerevisiae*. Several directed evolution experiments have been attempted by error prone PCR using HRPLs from *Pleurotus ostreatus *[46-48]. The results confirmed a general improvement in the total activity but poor secretion limits their practical engineering for other purposes. We recently described for the first time how to engineer HRPLs that can be expressed strongly by *S. cerevisiae*, enhancing their activities and thermostabilities [21]. Two different HRPLs were used to achieve this goal, the laccase from basidiomycete PM1 [24] and the laccase from *Pycnoporus cinnabarinus* (PcL) [23]. Several fusion proteins were tested by replacing the native signal sequences with others used successfully during heterologous expression in yeast. The best result was obtained with the α-factor prepro-leader and accordingly, the α-PM1 and α-PcL fusion proteins could then be subjected to the corresponding artificial evolution pathways. Interestingly, the joint evolution of the α-factor prepro-leader plus the HRPL allowed us to adjust each to the different protease specificities of the heterologous host throughout its transit from the endoplasmic reticulum to the Golgi compartment. For α-PM1 laccase, eight rounds of evolution were carried out in combination with rational approaches, Fig. (**4**). After screening over 50,000 clones generated by random mutagenesis, *in vivo* DNA shuffling, IvAM and site directed mutagenesis, the total laccase activity (which is the product of its secretion and kinetics) was enhanced up to 34,000-fold, the largest improvement ever reported for this kind of system [21]. We attribute such a large improvement to the joint evolution of the prepro-leader and the laccase, which meant that a synergistic effect was produced during its exportation by yeast. The ultimate variant obtained through this evolutionary process, the OB-1 mutant, was readily secreted by *S. cerevisiae* (up to 8 mg/L) in a soluble, very active and very stable form, particularly with respect to temperature (with a T_50_ value of 73ºC), pH and co-solvents, Fig. (**3B**). Among the strategies engineered to evolve HRPLs, the mutational exchange and the recovery of beneficial mutants should be highlighted. With mutational exchange, several mutations found in both evolutionary programmes (for the α-PM1 and the α-PcL) were switched from one system to another, taking advantage of their close sequence homology (above 75 %). Interestingly, some mutations found in the hydrophobic core of the α-factor pre-leader were valuable in both systems, which opens the possibility of evolving the α-factor prepro-leader as a universal signal peptide for the heterologous expression of fungal laccases in yeast. With mutational recovery, some beneficial mutations ruled out by the yeast recombination apparatus could be recovered by site-directed mutagenesis of OB-1 having mapped them first in the family tree of the whole evolution experiment. Last but not least, we paid special attention to protein stability since the overall philosophy of this work was to create a scaffold on which new functions could be developed and that was sufficiently stable to tolerate the introduction of a new set of beneficial mutations. Hence, the drops in stability produced during evolution by the accumulation of some beneficial but destabilizing mutations could be recovered by rational approaches, coupled with the screening of mutant libraries for thermostability, Fig. (**5**).

#### What have we Learnt?

Over the years, *S. cerevisiae* has helped us to engineer strategies for the directed evolution of fungal laccases. The low redox potential laccase MtL was the first successful example [41] and since then, we have designed specific approaches for the laboratory evolution of HRPLs that combine* in vivo* and *in vitro* tools with rational approaches [49]. The evolved fungal laccases mentioned in this review constitute unique platforms for further protein engineering through directed evolution, principally aimed at generating enzymes that can be used in attractive biotechnological applications. Indeed, we are now in a position to tailor HRPLs for different purposes, ranging from the engineering of 3D-nanobiodevices for analytical or biomedical use to the construction of cell factories in yeast [2, 49]. There are still some hurdles to overcome and some questions that should be answered: why is the functional expression of laccases in yeast so tremendously difficult? Would it be possible to evolve high-redox potential laccases in bacteria? Is it feasible to enhance the laccase redox potential beyond natural limits by directed evolution or by rational means without disturbing its stability?…and more significantly, would that improvement necessarily mean better activity? We hope that in the near future, new HRPLs engineered by directed evolution and rational approaches can affront the attractive challenges presented by traditional and modern biocatalysis.

## Figures and Tables

**Fig. (1):**
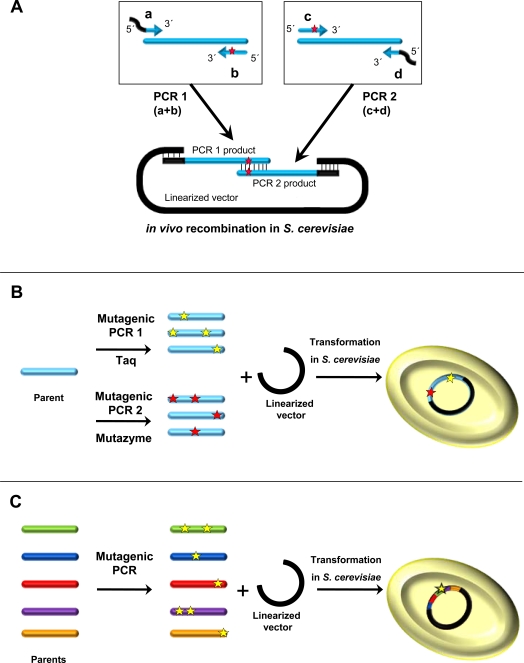
Different *in vivo* DNA recombination strategies based on the *S. cerevisiae* apparatus. **A**) IVOE; **B**) IvAM; **C**) *in vivo* DNA shuffling combined with mutagenic PCR.

**Fig. (3):**
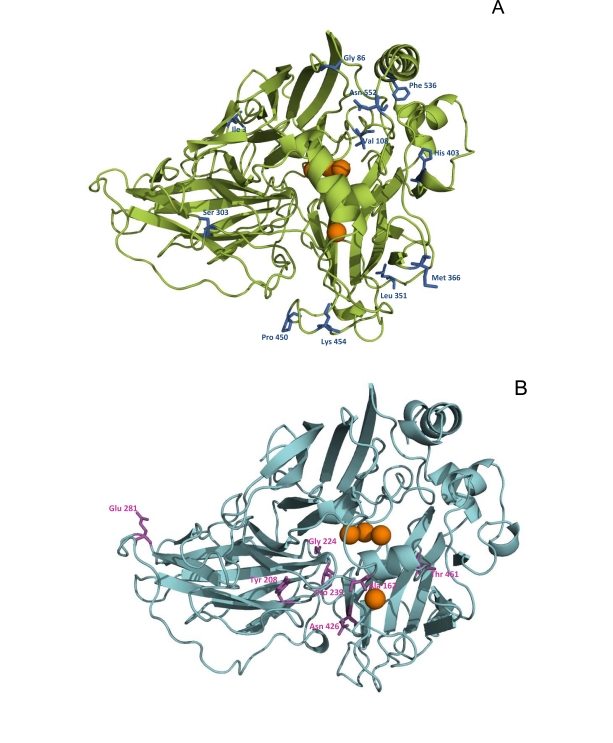
Amino acid substitutions accumulated in evolved laccases for functional expression in *S. cerevisiae.* **A**) MtLT2-variant; **B**) HRPLs PM1 (OB-1 variant). Orange spheres represent copper atoms. Amino acid substitutions found in mature MtLT2 and OB-1 are highlighted with stick structures.

**Fig. (4):**
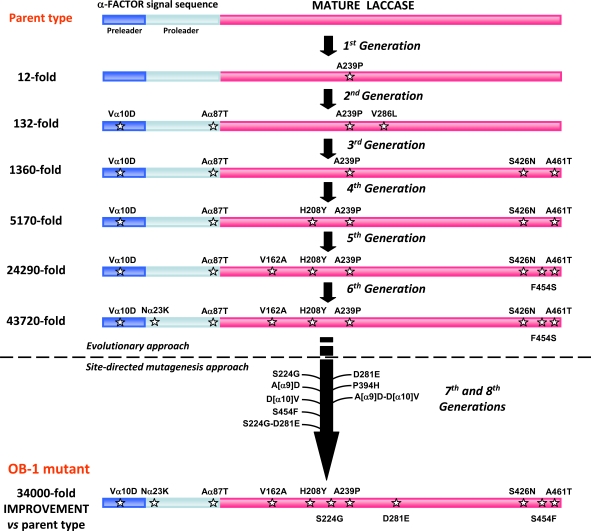
Combination of directed evolution and rational approaches for the engineering of HRPLs.

**Fig. (5):**
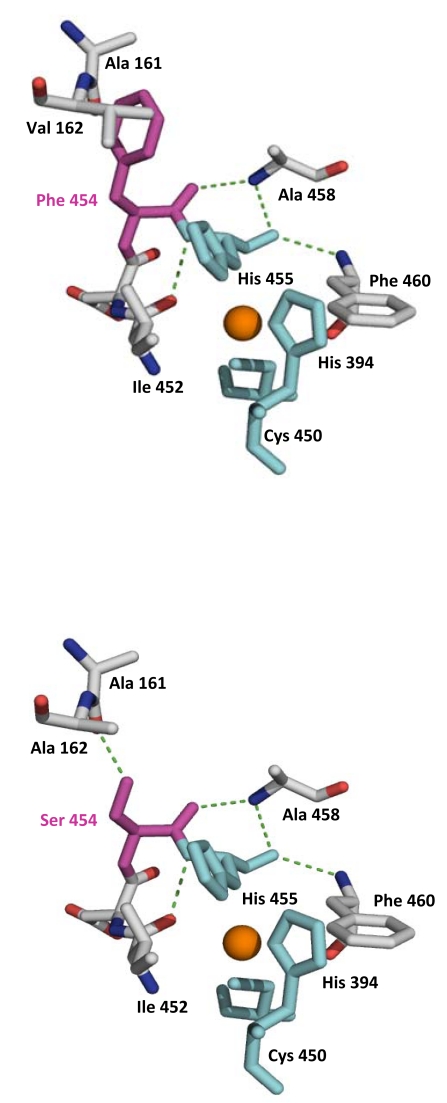
Rational approach for thermostability during the evolution of PM1 laccase. Mutation F454S discovered in the 5 ^th^ round enhanced the activity but at the cost of reducing the thermal stability (a hydrogen bond formed with Ala161 may affect the distance coordination between His455 and the T1 Cu). The reverted variant S454F completely recovered its stability which allowed the introduction of a new set of beneficial mutations.

## ABBREVIATIONS

ABTS: = 2,2-azino-bis[3-ethylbenzthizoline-6-sulfonic acid]
CLERY: = Combinatorial libraries enhanced by recombination in yeast
DMA: = Dimethyl acetamide
DMF: = Dimethyl formamide
DMP: = 2,6 dimethoxy phenol
DMSO: = Dimethyl sulfoxide
Eº_T1_: = Redox potential at the T1 site
HRPLs: = High redox potential laccases
IvAM: = *In vivo* assembly of mutant libraries
IVOE: = *In vivo* overlap extension
MtL: = *Myceliophthora thermophila* laccase
PAH: = Polycyclic aromatic hydrocarbon
PcL: = *Pycnoporus cinnabarinus* laccase
StEP: = Staggered extension process
T_50_: = The temperature at which the enzyme retains 50 % of its activity after a 10 min incubation
α-PcL: = Fusion gene of the α-factor prepro-leader and mature PcL
α-PM1: = Fusion gene of the α-factor prepro-leader and mature PM1 laccase
